# The inflammatory index and cytokines are associated with non-alcoholic fatty liver disease in type 2 diabetes mellitus

**DOI:** 10.3389/fmed.2025.1659998

**Published:** 2025-11-26

**Authors:** Ruiqing Liu, Ruiyan Liu, Mingjuan Liu, Yaqiong Tian, Jie Liu, Yao Wang, Qiangjun Sui, Jiandong Zhang, Hongmin Xu, Zhi Qi

**Affiliations:** 1 Central Hospital, Tianjin University/Tianjin Third Central Hospital, Tianjin Key Laboratory of Extracorporeal Life Support for Critical Diseases, Tianjin Artificial Cell Engineering Technology Research Center, Tianjin Institute of Hepatobiliary Disease, Tianjin, China; 2 Economic and Technological Development Zone Branch of Yueyang Public Security Bureau, Yueyang, Hunan, China; 3 Department of Medical Laboratory, Fenyang College, Shanxi Medical University, Fenyang, Shanxi, China; 4 Department of Molecular Pharmacology, School of Medicine, Nankai University, Tianjin, China

**Keywords:** NAFLD, T2DM, MHR, NLR, IL-6, TyG

## Abstract

**Background:**

Non-alcoholic fatty liver disease (NAFLD), which is characterized by hepatic steatosis in the absence of excessive alcohol consumption, is now increasingly recognized as a significant crucial factor contributing to chronic diseases, including diabetes. Moreover, it may progress to advanced hepatic pathologies such as fibrosis, cirrhosis, and even liver cancer. Systemic inflammation could be a potential mediator in the pathogenesis of diabetes secondary to NAFLD. Thus, we aim to evaluate inflammatory biomarkers to delineate their prognostic utility.

**Methods:**

A retrospective analysis was conducted on the clinical data of 624 participants from Tianjin Third Central Hospital, spanning from January 2023 to December 2024. Among them, 234 patients with NAFLD and type 2 diabetes mellitus (T2DM) were enrolled as NAFLD + T2DM group, 197 patients with T2DM were included in T2DM group and 193 healthy individuals were recruited into the control group. Independent t-tests or Mann–Whitney U tests were employed to compare demographic and biochemical parameters. Correlation analysis was carried out to assessed the association between NAFLD-T2DM comorbidity and systemic inflammation. The receiver operating characteristic curve (ROC) analysis was utilized to identify the optimal predictor and the optimum cut-off value for the comorbidity of NAFLD- and T2DM.

**Results:**

Among serum cytokines, laboratory indicators, and six indexes, TyG, MHR, NHR, NLR and IL-6 presented a significant positive correlation with the incidence in participants with NAFLD and T2DM. Additionally, NLR (AUC: 0.868) and IL-6 (AUC: 0.777) performed the best among inflammatory indicators and cytokines. The predictors obtained from the combined testing of NLR, IL-6, and TyG offer a superior predictive value for the identification and management of NAFLD in T2DM patients.

**Conclusion:**

Based on the findings, the predictors obtained from the combined testing of NLR, IL-6, and TyG emerge as the most practical and readily accessible indicators for early screening of NAFLD from patients with T2DM.

## 1 Introduction

Non-alcoholic fatty liver disease (NAFLD), a prevalent chronic liver condition characterized by excessive lipid accumulation (typically defined as >5% hepatocyte fat content) in the absence of significant alcohol consumption, poses a substantial global health burden intimately linked to metabolic dysfunction (1). The diagnosis of this lipid deposition relies on invasive methods like liver biopsy or non-invasive imaging techniques (2). Driven largely by the parallel rise in global obesity rates, the prevalence of NAFLD has surged dramatically, now affecting approximately 25% of the global population and accounting for 32.4% of all chronic liver disease cases (1). Epidemiological studies reveal substantial geographical disparities, with the highest disease burden observed in the Middle East (31.8%) and South America (30.5%), in contrast to the lowest prevalence in Africa (13.5%) (2). Notably, the morbidity of NAFLD in China escalates from 15% to over 40% in the 21st century, placing it among the most affected regions (3, 4). An alarming trend is the shifting demographic, as rising incidence rates among children and adolescents indicate a progressively younger trend of onset globally (5). Nowadays, with the prevalence of overweight and metabolic syndrome, the risk of NAFLD is approaching 75% in individuals with obesity and diabetes (1, 6). The clinical progression from simple steatosis (NAFL) to steatohepatitis (NASH) and subsequent fibrosis represents a critical stage in the poor prognosis, substantially elevating risks for end-stage liver complications, including hepatic decompensation, hepatocellular carcinoma (HCC), and liver failure necessitating transplantation (7). However, the often insidious and asymptomatic nature of early NAFLD pathogenesis frequently leads to under diagnosis and a lack of public awareness. Therefore, the early identification and proactive management of NAFLD are of paramount clinical importance.

Due to the interaction of multiple factors, NAFLD has a complex and varied pathogenesis. Adipose tissue dysfunction is a pivotal driver, which subsequently trigger impaired adipose expandability and leads free fatty acid overflow into the systemic circulation, ultimately promoting ectopic lipid deposition in hepatocytes (8). The classical “two-hit model” provides a foundational mechanism of NAFLD. The first hit suggests that abnormal lipid metabolism and insulin resistance (IR), which cause lipid accumulation in liver cells and lead to dysfunction in triglyceride synthesis and transport, thereby initiating NAFLD (9). A subsequent hit of oxidative stress-mediated lipid peroxidation triggers inflammatory cell infiltration, accelerating hepatocyte injury, fibro genesis, and necrosis (10, 11). An alternative hypothesis, termed the “multiple parallel hits” model, has been proposed to provide a more comprehensive explanation for the molecular pathways and metabolic regulation involved in NAFLD progression. This model encompasses ectopic fat accumulation, IR, oxidative stress (OS), endoplasmic reticulum (ER) stress (ERS), disrupted lipid metabolism, inflammation, and gut-microbiota dysfunction (12, 13).

Emerging evidence underscores a strong pathophysiological interplay between NAFLD and Type 2 diabetes mellitus (T2DM), driven largely by the shared mechanisms of IR and obesity (14, 15). T2DM, a chronic metabolic disorder characterized by hyperglycemia, exacerbates hepatic lipid metabolism dysregulation through IR. Specifically, IR fosters a hepatic inflammatory microenvironment by impairing adipocytes lipolysis and enhancing *de novo* lipogenesis in the liver, thereby accelerating the processes of hepatic inflammation, fibrosis, and cell death (16). Epidemiologically, it is notable that 65.04% of T2DM patients develop NAFLD, while 43.63% of NAFLD patients are diagnosed with concurrent T2DM (14). NAFLD amplifies systemic metabolic dysregulation in T2DM by impairing hepatic insulin clearance and promoting gluconeogenesis, collectively further accelerating their synergistically detrimental progression (14, 15).

The intricate pathogenesis of NAFLD remains incompletely elucidated. A hallmark of this pathogenesis entails dysregulation of lipid and glucose metabolism along with the activation of inflammatory signaling cascades. Consistent with this inflammatory component, we previously observed decreased levels of serum lymphocytes and monocytes in patients with NAFLD. Moreover, characteristic dyslipidemia-specifically reduced serum levels of high-density lipoprotein cholesterol (HDL-C) and the increased levels of total cholesterol (TC), triglycerides (TG), and light-density lipoprotein cholesterol (LDL-C) were detected in patients with T2DM and NAFLD. Several studies have investigated the diagnostic value of the monocyte to HDL-C ratio (MHR) in NAFLD (17), limited research has been conducted on the diagnostic capacity of broader inflammatory indexes in this context. Therefore, this study aims to evaluate the association between a comprehensive panel of inflammatory indicators (including routine laboratory assessments, novel inflammatory indexes, and cytokine levels) and the risk of NAFLD in patients with T2DM. Additionally, this study seeks to evaluate the combined diagnostic performance of the triglyceride-glucose index (TyG) alongside these inflammatory indicators, aiming to better distinguish patients with T2DM and NAFLD from those with T2DM alone.

## 2 Materials and methods

### 2.1 Study design and population

Our study retrospectively analyzed the clinical data of patients diagnosed with hyperglycemia, along with a series of laboratory assessments conducted at the Tianjin Third Central Hospital from January 2023 to December 2024. Based on the current or past ultrasound examination, participants were categorized into two groups: T2DM and T2DM + NAFLD. Among the participants, 234 patients had T2DM accompanied by NAFLD, while 197 had T2DM without NAFLD. This cross-sectional included 193 healthy individuals who underwent health check-ups at the Tianjin Third Central Hospital.

### 2.2 Definition and measurement of NAFLD and T2DM

The diagnosis of T2DM was determined based on the criteria, which define fasting plasma glucose (FPG) > 7 mmol/L or 2 h postprandial plasma glucose (2hPG) > 11.1 mmol/L (18). The diagnostic criterion for NAFLD was based on the guidelines for prevention and treatment of NAFLD developed by the Chinese Society of Liver Diseases (19). NAFLD was diagnosed in patients presenting with at least two of the following three findings: (a) diffuse enhancement of the liver near-field echo that is stronger than that of the kidney, (b) poorly defined intrahepatic bile duct structures, and (c) gradual attenuation of the far-field echo of the liver. Participants may also exhibit nonspecific symptoms, such as vague pain in the liver area, fatigue, and hepatosplenomegaly.

To ensure the effectiveness of the results, comprehensive exclusion criteria were implemented as follows: (a) individuals with chronic excessive alcohol intake (defined as ≥210 g/week for males and ≥140 g/week for females); (b) individuals with acute or chronic infections; (c) patients with hematologic disorders including anemia, hemolytic diseases, bleeding, and other disorders which resulted in abnormal hemolytic status; (d) patients with lipid metabolism dysfunction; (e) patients with severe spinal curvature. Following rigorous screening, 197 patients with T2DM and 234 patients with T2DM comorbid with NAFLD were included in the final cohort.

### 2.3 Data collection and laboratory measurements

The clinical data were retrospectively extracted from the electronic medical record system and categorized into three domains: (a) demographic characteristics: age and gender (b) laboratory parameters: complete blood count parameters [including white blood cells (WBC), neutrophils (NEU), lymphocytes (LYM), monocytes (MONO) and platelets (PLT)], FPG, albumin (ALB), total protein (TP), total cholesterol (TC), triglycerides (TG), high-density lipoprotein cholesterol (HDL-C) and low-density lipoprotein cholesterol (LDL-C), alanine aminotransferase (ALT), aspartate aminotransferase (AST), gamma-glutamyl transferase (GGT), C reaction protein (CRP), ferritin (FER) and procalcitonin (PCT). Venous blood samples were collected following a standardized 12 h overnight fast. (c) In the process of data statistics, stratified sampling was employed to conduct random selection among the participants. The criteria for stratified sampling were the patients’ glycosylated hemoglobin index and blood glucose values. Whole blood samples were centrifuged at 1,500 rpm for 20 min. Serum cytokines (IL-2, IL-4, IL-6, IL-10, IL-17, IFN-γ, and TNF-α) were quantified using a flow cytometer (FACS Canto II, BD Biosciences, Inc.).

### 2.4 Six indirect indexes

The values of triglyceride-glucose index (TyG), MHR, NHR, NLR, PWR and Systemic immune-inflammatory index (SII) were derived from the laboratory measurements using the following Equations 1–6


(1) TyG=LN[TG×FPG/2]

(2) $$\mathrm{MHR}=\frac{\text{MONO}}{\mathrm{HDL}-C}$$

(3) $$\mathrm{NHR}=\frac{\mathrm{NEU}}{\mathrm{HDL}-C}$$

(4) $$\mathrm{NLR}=\frac{\mathrm{NEU}}{\mathrm{LYM}}$$

(5) $$\mathrm{PWR}=\frac{\mathrm{PLT}}{\mathrm{WBC}}$$

(6) $$\mathrm{SII}=\frac{\mathrm{NEU}\times \mathrm{PLT}}{\mathrm{LYM}}$$

### 2.5 Statistical analysis

Statistical analyses were conducted using SPSS software (V 27; IBM Corp., Armonk, NY, USA) and GraphPad Prism 8 software (Graph Pad Software, Inc., La Jolla, CA, USA). Continuous variables with normal distribution were expressed as mean ± standard deviation (SD), while non-normally distributed variables were expressed as the median of the interquartile range (IQR) (25%, 75%).

Descriptive analyses were performed using Student’s *t*-tests or Mann–Whitney *U* test based on distribution characteristics. Spearman correlation analysis was constructed to assess the association between each biomarker. Additionally, receiver operating characteristic (ROC) curve analysis evaluated predictive capacity and diagnostic accuracy. Optimal cut-off values were determined using Youden’s index maximization criteria. Odds ratios (OR) and their corresponding 95% confidence intervals of combined diagnostic prediction are calculated through binary logistic regression analysis. Use the Bonferroni corrected *p*-value and set a *p*-value <0.05 as statistically significant.

## 3 Results

### 3.1 General clinical characteristics of the participants

This cross-sectional study enrolled a cohort of 624 adults (341 males; 283 females) aged 21–84 years. Based on clinical characteristics, the population comprised three distinct groups: 197 individuals diagnosed with T2DM, 234 with T2DM and NAFLD, and 193 healthy controls. Table 1 summarizes the characteristics of age, ALT, AST, GGT, FPG, ALB, and TP across study groups, expressed as the mean (SD)/IQR. The indicators in the control group were 46.0 (11.8), 15 (10, 18), 18 (14, 21), 19 (14, 29.5), 5.12 (4.93, 5.43), 46.55 (2.43), and 72.37 (3.73), the T2DM cohort were 58.4 (12.7), 18 (13, 30), 17 (14, 25.5), 26 (16, 52), 8.76 (7.67, 9.94), 40.28 (4.05), and 64.79 (5.22), and the T2DM and NAFLD group indicators were 59.7 (10.5), 22.5 (15, 36), 30 (20, 38.25), 48 (26.75, 91), 8.84 (7.41, 10.86), 35.75 (5.95), and 64.17 (8.24).

**Table 1:** General clinical characteristics of the participants.

Outcomes	Control (*n* = 193)	T2DM (*n* = 197)	T2DM + NAFLD (*n* = 234)	*p^1^-*value	*p^2^-*value	*p^3^-*value
Mean age, years, mean (s.d.)	46.0 (11.8)	58.4 (12.7)	59.7 (10.5)	**<0.0001**	**<0.0001**	0.93
ALT, U/L, median (Q1, Q3)	15 (10, 18)	18 (13, 30)	22.5 (15, 36)	**<0.0001**	**<0.0001**	0.3
AST, U/L, median (Q1, Q3)	18 (14, 21)	17 (14, 25.5)	30 (20, 38.25)	**<0.01**	**<0.0001**	**<0.0001**
GGT, U/L, median (Q1, Q3)	19 (14, 29.5)	26 (16, 52)	48 (26.75, 91)	**<0.0001**	**<0.0001**	**<0.0001**
FPG, mmol/L, median (Q1, Q3)	5.12 (4.93, 5.43)	8.76 (7.67, 9.94)	8.84 (7.41, 10.86)	**<0.0001**	**<0.0001**	**<0.0001**
ALB, g/L, mean (s.d.)	46.55 (2.43)	40.28 (4.05)	35.75 (5.95)	**<0.0001**	**<0.0001**	**<0.0001**
TP, g/L, mean (s.d.)	72.37 (3.73)	64.79 (5.22)	64.17 (8.24)	**<0.0001**	**<0.0001**	0.5

Data were shown as means ± SD or medians with interquartile ranges (IQRs). ALT, alanine aminotransferase; AST, aspartate aminotransferase; GGT, gamma-glutamyl transferase; FPG, fasting plasma glucose; ALB, albumin; TP, total protein. *p^1^*: control group vs. T2DM group; *p^2^*: control group vs. T2DM + NAFLD group; *p^3^*: T2DM group vs. T2DM + NAFLD group. *p*-value <0.05 is considered significant. Bold values represent statistically significant results.

Significant intergroup differences in baseline characteristics were observed between cohorts with and without NAFLD comorbidity in the T2DM population, except for age, ALT, and TP. In comparison, the participants with NAFLD comorbidity tended toward advanced age and marginally elevated ALT levels. Additionally, the T2DM + NAFLD cohort exhibited clinically significant hepatic biomarker alterations, including elevated AST, GGT, and reduced ALB. These pathophysiological changes collectively suggest progressive hepatic metabolic dysregulation in the comorbid population.

### 3.2 Lipids laboratory indicators of the participants

Table 2 showed statistically different lipid metabolism characteristics between cohorts with and without T2DM or NAFLD comorbidity, except for LDL-C (control *vs.* T2DM) and TyG index (T2DM *vs.* T2DM + NAFLD). Meanwhile the participants with NAFLD comorbidity tended to have an advanced TyG index. It is worth noting that participants with T2DM and NAFLD exhibited statistically increased levels of TC, TG, LDL-C, TyG and reduced level of HDL-C. Moreover, compared with the control group, the levels of TC, TG, LDL-C, and TyG in T2DM individuals were higher, while the HDL-C level was lower. These findings indicate that the participants with T2DM and NAFLD have hyperlipidemia.

**Table 2:** Lipids laboratory indicators of the participants.

Outcomes	Control (*n* = 193)	T2DM (*n* = 197)	T2DM + NAFLD (*n* = 234)	*p^1^-*value	*p^2^-*value	*p^3^-*value
TC, mmol/L, mean (s.d.)	4.76 (0.83)	4.59 (1.05)	4.92 (1.72)	**<0.0001**	**<0.0001**	**<0.05**
TG, mmol/L, median (Q1, Q3)	1.18 (0.87, 1.72)	1.78 (1.24, 2.29)	2.15 (1.39, 2.73)	**<0.0001**	**<0.0001**	**<0.05**
LDL-C, mmol/L, mean (s.d.)	2.66 (0.56)	2.68 (0.68)	3.06 (1.22)	0.73	**<0.0001**	**<0.001**
HDL-C, mmol/L, median (Q1, Q3)	1.22 (1.02, 1.41)	0.94 (0.84, 1.09)	0.88 (0.71, 1.07)	**<0.0001**	**<0.0001**	**<0.05**
TyG, median (Q1, Q3)	1.12 (0.79, 1.59)	2.09 (1.73, 2.44)	2.23 (1.79, 2.51)	**<0.0001**	**<0.0001**	0.51

Data were shown as means ± SD or medians with interquartile ranges (IQRs). HDL-C, high-density lipoprotein cholesterol; LDL-C, light-density lipoprotein cholesterol; TC, total cholesterol; TG, triglycerides; TyG, triglyceride-glucose, TyG = LN[TG(mg/dL) × FPG(mg/dL)/2]. *p^1^*: control group vs. T2DM group; *p^2^*: control group vs. T2DM + NAFLD group; *p^3^*: T2DM group vs. T2DM + NAFLD group. *p*-value <0.05 is considered significant. Bold values represent statistically significant results.

### 3.3 Inflammatory characteristics of the participants

The SD and IQR of inflammatory characteristics variables are shown in Table 3. Compared to the control group, the T2DM cohort demonstrated increased levels of NEU, MONO, CRP, FER, NLR, MHR, NHR, and SII, alongside decreased levels of LYM, PLT, PCT, and PWR. Notably, there was no significant difference in WBC between the two groups. Additionally, Compared to the control group, the T2DM and NAFLD cohort exhibited elevated levels of NEU, CRP, FER, NLR, MHR, NHR and SII couple with attenuated levels of WBC, PLT, PCT and PWR. Furthermore, comparative analysis between the T2DM cohort and the T2DM + NAFLD comorbid cohort revealed intensified inflammatory activity in comorbid patients with higher levels of CRP, FER, NLR, and MHR. While the levels of NHR and SII in the comorbid cohort were lower than in the T2DM cohort. We hypothesized that these results may be related to lower level of WBC in participants with T2DM and NAFLD. These hematologic characteristics may reflect compensatory immunosuppression mechanisms in progressive metabolic.

**Table 3:** Inflammatory characteristics of the participants.

Outcomes	Control (*n* = 193)	T2DM (*n* = 197)	T2DM + NAFLD (*n* = 234)	*p^1^-*value	*p^2^-*value	*p^3^-*value
WBC, 10^9^/L, median (Q1, Q3)	6.68 (5.47, 7.67)	6.7 (5.79, 8.07)	4.95 (3.07, 7.08)	0.13	**<0.0001**	**<0.0001**
NEU, 10^9^/L, mean (s.d.)	3.84 (1.24)	4.33 (1.34)	3.86 (2.25)	**<0.001**	0.92	**<0.05**
LYM, 10^9^/L, median (Q1, Q3)	2.21 (1.85, 2.64)	1.82 (1.44, 2.21)	0.84 (0.53, 1.25)	**<0.0001**	**<0.0001**	**<0.0001**
MONO, 10^9^/L, median (Q1, Q3)	0.37 (0.31, 0.45)	0.42 (0.33, 0.52)	0.36 (0.21, 0.46)	**<0.0001**	0.40	**<0.001**
PLT, 10^9^/L, mean (s.d.)	246.81 (52.15)	218.42 (66.41)	116.6 (76.73)	**<0.0001**	**<0.0001**	**<0.0001**
CRP, mg/L, median (Q1, Q3)	1.19 (0.61, 1.67)	4.6 (2.64, 9.73)	5.28 (19.65)	**<0.001**	**<0.0001**	0.06
FER, ng/mL, median (Q1, Q3)	2.17 (1.7, 2.49)	18.3 (14.0, 26.9)	159.36 (64.1, 241.9)	**<0.0001**	**<0.0001**	**<0.0001**
PCT, ng/mL, median (Q1, Q3)	0.23 (0.21, 0.26)	0.22 (0.17, 0.26)	0.08 (0.05, 0.1)	**<0.01**	**<0.0001**	**<0.0001**
NLR, median (Q1, Q3)	1.65 (1.22, 2.06)	2.27 (1.76, 3.04)	4.10 (2.82, 5.94)	**<0.0001**	**<0.0001**	**<0.0001**
PWR, median (Q1, Q3)	37.03 (30.74, 44.75)	31.37 (25.01, 39.01)	21.25 (15.08, 29.58)	**<0.0001**	**<0.0001**	**<0.0001**
MHR, median (Q1, Q3)	0.31 (0.24, 0.38)	0.41 (0.33, 0.49)	0.43 (0.33, 0.62)	**<0.0001**	**<0.0001**	**<0.001**
NHR, median (Q1, Q3)	3.1 (2.15, 4.06)	4.46 (3.56, 5.87)	4.14 (2.63, 5.91)	**<0.0001**	**<0.0001**	0.96
SII, median (Q1, Q3)	394.81 (278.29, 504.45)	466.07 (358.68, 643.79)	436.68 (240.35, 714.37)	**<0.0001**	**<0.0001**	0.34

Data were shown as means ± SD or medians with interquartile ranges (IQRs). WBC, white blood cell; NEU, neutrophil; LYM, lymphocyte; MONO, monocyte; PLT, platelet; CRP, C reaction protein; FER, ferritin; PCT, procalcitonin‌; NLR = NEU/LYM; PWR = PLT/WBC; MHR = MONO/HDL-C; NHR = NEU/HDL-C; SII = (NEU×PLT)/LYM. *p^1^*: Control group vs. T2DM group; *p^2^*: control group vs. T2DM + NAFLD group; *p^3^*: T2DM group vs. T2DM + NAFLD group. *p*-value <0.05 is considered significant. Bold values represent statistically significant results.

### 3.4 The cytokines indicators of the participants

**Figure 1:**
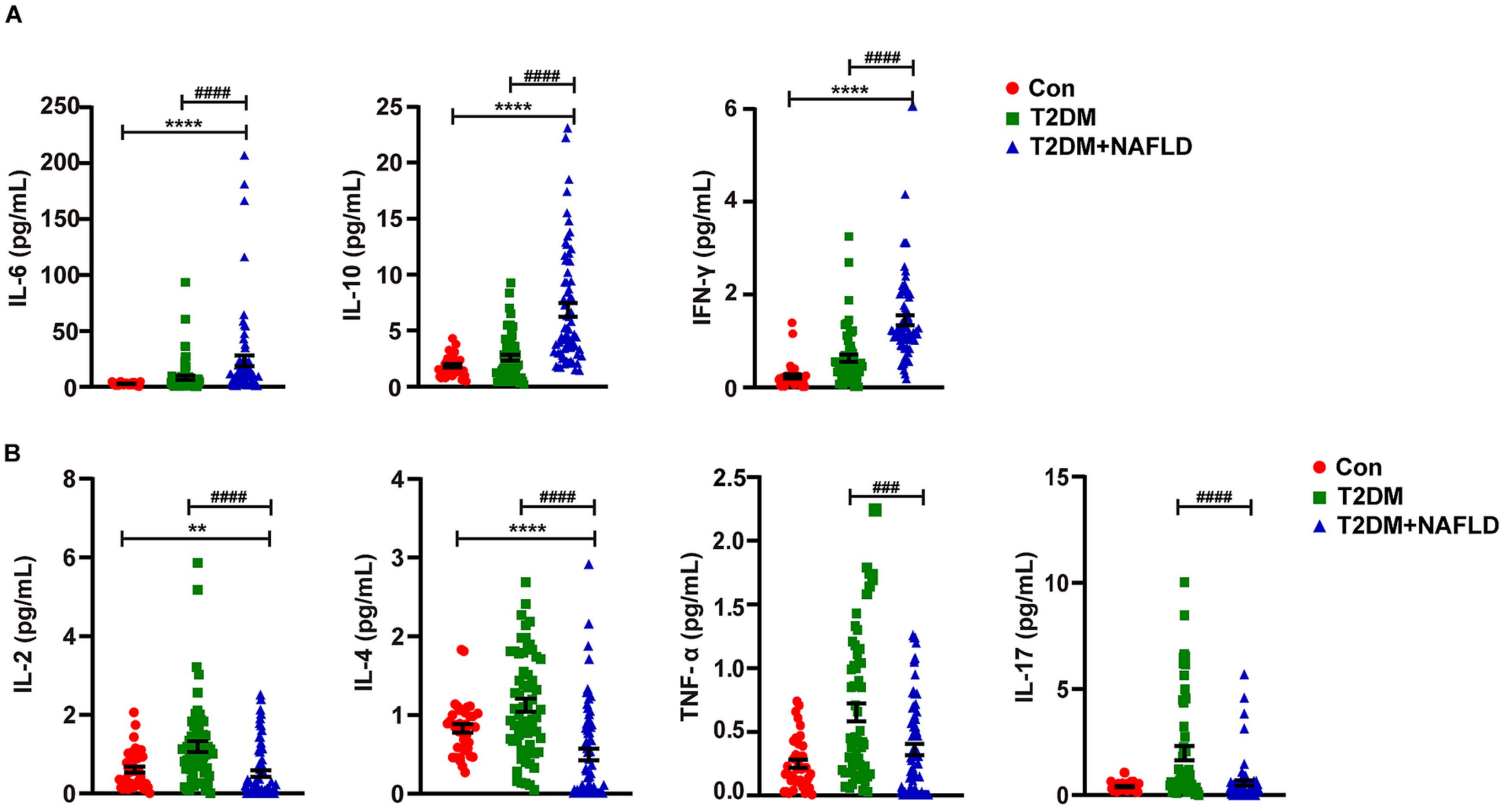
Detection of the serum cytokines using flow cytometry. **(A)** Serum IL-6, IL-10 and IFN-γ were detected by Flow cytometry. **(B)** Serum IL-2, IL-4, IL-17 and TNF-α were detected by Flow cytometry. Data shown are means ± SEMs; ^**^p < 0.01, ^****^p < 0.0001 (NAFLD + T2DM group vs. control group); ^###^*p* < 0.001, ^####^*p* < 0.0001 (NAFLD + T2DM group vs. T2DM group).

To further delineate the inflammatory spectrum in NAFLD progression, a stratified random sampling approach was employed. The distribution trends within the subsample were similar to those of the overall sample. Subsequently cytokines profiling was systematically conducted. As detailed in Table 4, the T2DM + NAFLD cohort demonstrated statistically elevated concentrations of IL-6, IL-10, and IFN-γ compared to mono disease cohorts, whereas the concentrations of IL-2, IL-4, and IL-17 were reduced. Additionally, the expression levels of differential cytokines across clinical subgroups were exhibited more clearly in Figure 1, which revealed distinct inflammatory signatures associated with disease progression.

**Table 4:** The cytokines indicators of the participants.

Outcomes	Control (*n* = 40)	T2DM (*n* = 61)	T2DM + NAFLD (*n* = 69)	*p^1^-*value	*p^2^-*value	*p^3^-*value
IL-2, pg/mL, median (Q1, Q3)	0.55 (0.23, 0.93)	1.01 (0.57, 1.49)	0.13 (0.01, 0.77)	**<0.001**	**<0.01**	**<0.0001**
IL-4, pg/mL, median (Q1, Q3)	0.85 (0.58, 1.02)	1.07 (0.65, 1.73)	0.22 (0.01, 0.87)	**<0.01**	**<0.0001**	**<0.0001**
IL-6, pg/mL, median (Q1, Q3)	2.41 (1.56, 3.39)	2.44 (1.34, 6.83)	10.36 (4.04, 21.96)	0.32	**<0.0001**	**<0.0001**
IL-10, pg/mL, median (Q1, Q3)	1.56 (1.28, 2.35)	1.95 (1.14, 3.62)	4.62 (3.02, 9.4)	0.39	**<0.0001**	**<0.0001**
TNF-α, pg/mL, median (Q1, Q3)	0.21 (0.11, 0.37)	0.42 (0.18, 1.04)	0.25 (0.03, 0.62)	**<0.001**	0.75	**<0.001**
IFN-γ, pg/mL, median (Q1, Q3)	0.17 (0.12, 0.25)	0.45 (0.25, 0.77)	1.19 (0.89, 1.98)	**<0.0001**	**<0.0001**	**<0.0001**
IL-17, pg/mL, median (Q1, Q3)	0.34 (0.25, 0.54)	0.74 (0.38, 2.75)	0.25 (0.04, 0.67)	**<0.0001**	0.12	**<0.0001**

Data were shown as medians with interquartile ranges (IQRs). *p^1^*: control group vs. T2DM group; *p^2^*: control group vs. T2DM + NAFLD group; *p^3^*: T2DM group vs. T2DM + NAFLD group. *p*-value <0.05 is considered significant. Bold values represent statistically significant results.

### 3.5 Spearman correlation analysis of TyG, MHR, NHR, NLR, SII, cytokines, and laboratory indicators

Table 5 illustrates the correlation between the TyG index and the NAFLD-related inflammatory indicators (WBC: *r* = 0.136, *p* = 0.011; NEU: *r* = 0.246, *p* = 0.0011; CRP: *r* = 0.373, *p* = 0.0011; FER: *r* = 0.541, *p* = 0.0011; MHR: *r* = 0.366, *p* = 0.0011; NHR: *r* = 0.441, *p* = 0.0011; NLR: *r* = 0.319, *p* = 0.0011; SII: *r* = 0.143, *p* = 0.011; IFN-γ: *r* = 0.229, *p* = 0.088). The findings revealed a statistically positive association between elevated TyG index and an aggravated inflammation of NAFLD.

**Table 5:** Spearman correlation analysis in TyG and inflammatory indicators.

Outcomes	TyG			
	*r*	*p*-value	Bonferroni-adjusted *p*-value	Significant
WBC, 10^9^/L	0.136	**0.001**	**0.011**	**YES**
NEU, 10^9^/L	0.246	**<0.0001**	**0.0011**	**YES**
CRP	0.373	**<0.0001**	**0.0011**	**YES**
FER	0.541	**<0.0001**	**0.0011**	**YES**
MHR	0.366	**<0.0001**	**0.0011**	**YES**
NHR	0.441	**<0.0001**	**0.0011**	**YES**
NLR	0.319	**<0.0001**	**0.0011**	**YES**
SII	0.143	**0.001**	**0.011**	**YES**
IL-6	0.14	0.108	1.000	NO
IL-10	0.095	0.278	1.000	NO
IFN-γ	0.229	**0.008**	0.088	NO

TyG = LN[TG(mg/dL) × GLU(mg/dL)/2]; WBC, white blood cell; Neu, neutrophil; CRP, C reaction protein; FER, ferritin; MHR = MONO/HDL-C; NHR = NEU/HDL-C; NLR = NEU/LYM; SII = (NEU×PLT)/LYM. *p*-value <0.05 is considered significant.

Additionally, we analyzed the correlation between the inflammatory indexes (MHR, NHR, NLR, SII) and generation laboratory indicators. Tables 6–9 shows that MHR has a positive association with WBC (*r* = 0.373, *p* < 0.001), ALT (*r* = 0.227, *p* < 0.001), AST (*r* = 0.227, *p* < 0.001), GGT (*r* = 0.295, *p* < 0.001), FPG (*r* = 0.244, *p* = 0.036) and TG (*r* = 0.303, *p* < 0.001). NHR positively associates with WBC (*r* = 0.699, *p* < 0.001), ALT (*r* = 0.185, *p* < 0.001), GGT (*r* = 0.229, *p* < 0.001), FPG (*r* = 0.198, *p* < 0.001), TG (*r* = 0.408, *p* < 0.001), and LDL-C (*r* = 0.133, *p* = 0.036). NLR positively correlates with age (*r* = 0.304, *p* < 0.001), ALT (*r* = 0.25, *p* < 0.001), AST (*r* = 0.221, *p* < 0.001), GGT (*r* = 0.263, *p* < 0.001), FPG (*r* = 0.191, *p* < 0.001), and TG (*r* = 0.191, *p* < 0.001). SII has a positive association with WBC (*r* = 0.462, *p* < 0.001) and LDL-C (*r* = 0.156, *p* < 0.001) and a negative association with AST (*r* = −0.152, *p* = 0.108). Among them, NHR presented a superior positive association with generation laboratory indicators.

**Table 6:** Spearman correlation analysis in MHR and generation laboratory indicators.

Outcomes	MHR			
	*r*	*p-*value	Bonferroni-adjusted *p*-value	Significant
Age, years	0.099	**0.017**	0.612	NO
WBC, 10^9^/L	0.373	**<0.0001**	**<0.001**	**YES**
ALT, U/L	0.227	**<0.0001**	**<0.001**	**YES**
AST U/L	0.227	**<0.0001**	**<0.001**	**YES**
GGT, U/L	0.295	**<0.0001**	**<0.001**	**YES**
FPG, mmol/L	0.244	**<0.001**	**0.036**	**YES**
TC, mmol/L	0.063	0.132	1	NO
TG, mmol/L	0.303	**<0.0001**	**<0.001**	**YES**
LDL-C, mmol/L	0.091	**0.029**	1	NO

MHR = MONO/HDL-C; WBC, white blood cell; ALT, alanine aminotransferase; AST, aspartate aminotransferase; GGT, gamma-glutamyl transferase; FPG, fasting plasma glucose; LDL-C, light-density lipoprotein cholesterol; TC, total cholesterol; TG, triglycerides. *p*-value <0.05 is considered significant. Bold values represent statistically significant results.

**Table 7:** Spearman correlation analysis in NHR and generation laboratory indicators.

Outcomes	NHR			
	*r*	*p-*value	Bonferroni-adjusted *p*-value	Significant
Age, years	0.068	0.102	1	NO
WBC, 10^9^/L	0.699	**<0.0001**	**<0.001**	**YES**
ALT, U/L	0.185	**<0.0001**	**<0.001**	**YES**
AST U/L	0.037	**<0.0001**	**<0.001**	**YES**
GGT, U/L	0.229	**<0.0001**	**<0.001**	**YES**
FPG, mmol/L	0.198	**<0.0001**	**<0.001**	**YES**
TC, mmol/L	0.067	0.106	1	NO
TG, mmol/L	0.408	**<0.0001**	**<0.001**	**YES**
LDL-C, mmol/L	0.133	**0.001**	**0.036**	**YES**

NHR = NEU/HDL-C; WBC, white blood cell; ALT, alanine aminotransferase; AST, aspartate aminotransferase; GGT, gamma-glutamyl transferase; FPG, fasting plasma glucose; LDL-C, light-density lipoprotein cholesterol; TC, total cholesterol; TG, triglycerides. *p*-value <0.05 is considered significant. Bold values represent statistically significant results.

**Table 8:** Spearman correlation analysis in NLR and generation laboratory indicators.

Outcomes	NLR			
	*r*	*p-*value	Bonferroni-adjusted *p*-value	Significant
Age, years	0.304	**<0.0001**	**<0.001**	**YES**
WBC, 10^9^/L	−0.002	0.962	1	NO
ALT, U/L	0.25	**<0.0001**	**<0.001**	**YES**
AST U/L	0.221	**<0.0001**	**<0.001**	**YES**
GGT, U/L	0.263	**<0.0001**	**<0.001**	**YES**
FPG, mmol/L	0.179	**<0.0001**	**<0.001**	**YES**
TC, mmol/L	0.011	0.782	1	NO
TG, mmol/L	0.191	**<0.0001**	**<0.001**	**YES**
LDL-C, mmol/L	0.099	**0.017**	0.612	NO

NLR = NEU/LYM; WBC, white blood cell; ALT, alanine aminotransferase; AST, aspartate aminotransferase; GGT, gamma-glutamyl transferase; FPG, fasting plasma glucose; LDL-C, light-density lipoprotein cholesterol; TC, total cholesterol; TG, triglycerides. *p*-value <0.05 is considered significant. Bold values represent statistically significant results.

**Table 9:** Spearman correlation analysis in SII and generation laboratory indicators.

Outcomes	SII			
	*r*	*p-*value	Bonferroni-adjusted *p*-value	Significant
Age, years	0.066	0.107	1	NO
WBC, 10^9^/L	0.462	**<0.0001**	**<0.001**	**YES**
ALT, U/L	0.017	**<0.0001**	**<0.001**	**YES**
AST U/L	−0.152	**0.003**	0.108	NO
GGT, U/L	−0.012	0.825	1	NO
FPG, mmol/L	0.079	0.056	1	NO
TC, mmol/L	0.067	0.107	1	NO
TG, mmol/L	0.087	**0.037**	1	NO
LDL-C, mmol/L	0.156	**<0.0001**	**<0.001**	**YES**

SII = (NEU×PLT)/LYM; WBC, white blood cell; ALT, alanine aminotransferase; AST, aspartate aminotransferase; GGT, gamma-glutamyl transferase; FPG, fasting plasma glucose; LDL-C, light-density lipoprotein cholesterol; TC, total cholesterol; TG, triglycerides. *p*-value <0.05 is considered significant. Bold values represent statistically significant results.

To further determine the relationship between NAFLD and inflammation, we chose IL-6, IL-10 and IFN-γ to participate in this analysis, which had higher expression levels in the participants with T2DM and NAFLD. We found a positive statistical correlation between IL-6 and AST (*r* = 0.248, *p* = 0.027), GGT (*r* = 0.339, *p* = 0.0027), and a negative correlation between IL-6 and WBC (*r* = −0.323, *p* = 0.0027). Furthermore, IL-10 revealed a positive correlation with AST (*r* = 0.246, *p* = 0.027) and GGT (*r* = 0.316, *p* = 0.0027). In addition, IL-10 and IFN-γ also have a negative correlation with WBC and HDL-C (Tables 10–12). These results suggested that elevated IL-6 concentrations significantly correlated with aggravated inflammation and lipid deposition.

**Table 10:** Spearman correlation analysis in IL-6 and generation laboratory indicators.

Outcomes	IL-6			
	*r*	*p-*value	Bonferroni-adjusted *p*-value	Significant
Age, years	0.199	**0.009**	0.243	NO
WBC, 10^9^/L	−0.323	**<0.0001**	**0.0027**	**YES**
ALT, U/L	0.178	**0.021**	0.567	NO
AST, U/L	0.248	**0.001**	**0.027**	**YES**
GGT, U/L	0.339	**<0.0001**	**0.0027**	**YES**
FPG, mmol/L	0.203	**0.009**	0.243	NO
TC, mmol/L	−0.121	0.123	1	NO
TG, mmol/L	0.073	0.372	1	NO
LDL-C, mmol/L	−0.147	0.083	1	NO
HDL-C, mmol/L	−0.173	**0.041**	1	NO

WBC, white blood cell; ALT, alanine aminotransferase; AST, aspartate aminotransferase; GGT, gamma-glutamyl transferase; FPG, fasting plasma glucose; HDL-C, high-density lipoprotein cholesterol; LDL-C, light-density lipoprotein cholesterol; TC, total cholesterol; TG, triglycerides. *p*-value <0.05 is considered significant. Bold values represent statistically significant results.

**Table 11:** Spearman correlation analysis in IL-10 and generation laboratory indicators.

Outcomes	IL-10			
	*r*	*p-*value	Bonferroni-adjusted *p*-value	Significant
Age, years	0.119	0.123	1	NO
WBC, 10^9^/L	−0.206	**0.007**	0.189	NO
ALT, U/L	0.240	**0.002**	0.054	NO
AST, U/L	0.246	**0.001**	**0.027**	**YES**
GGT, U/L	0.316	**<0.0001**	**0.0027**	**YES**
FPG, mmol/L	0.189	**0.016**	0.432	NO
TC, mmol/L	−0.024	0.756	1	NO
TG, mmol/L	0.096	0.238	1	NO
LDL-C, mmol/L	−0.083	0.331	1	NO
HDL-C, mmol/L	−0.075	0.38	1	NO

WBC, white blood cell; ALT, alanine aminotransferase; AST, aspartate aminotransferase; GGT, gamma-glutamyl transferase; FPG, fasting plasma glucose; HDL-C, high-density lipoprotein cholesterol; LDL-C, light-density lipoprotein cholesterol; TC, total cholesterol; TG, triglycerides. *p*-value <0.05 is considered significant. Bold values represent statistically significant results.

**Table 12:** Spearman correlation analysis in IFN-γ and generation laboratory indicators.

Outcomes	IFN-γ			
	*r*	*p-*value	Bonferroni-adjusted *p*-value	Significant
Age, years	0.194	**0.011**	0.297	NO
WBC, 10^9^/L	−0.163	**0.034**	0.918	NO
ALT, U/L	0.064	0.412	1	NO
AST, U/L	0.074	0.34	1	NO
GGT, U/L	0.15	0.054	1	NO
FPG, mmol/L	0.25	**0.001**	**0.027**	**YES**
TC, mmol/L	0.012	0.882	1	NO
TG, mmol/L	0.19	**0.019**	0.513	NO
LDL-C, mmol/L	−0.047	0.58	1	NO
HDL-C, mmol/L	−0.206	**0.015**	0.405	NO

WBC, white blood cell; ALT, alanine aminotransferase; AST, aspartate aminotransferase; GGT, gamma-glutamyl transferase; FPG, fasting plasma glucose; HDL-C, high-density lipoprotein cholesterol; LDL-C, light-density lipoprotein cholesterol; TC, total cholesterol; TG, triglycerides. *p*-value <0.05 is considered significant. Bold values represent statistically significant results.

### 3.6 ROC-based predictive modeling: diagnostic performance of hematologic ratios (MHR/NHR/NLR/SII) and cytokine profiling for T2DM-NAFLD comorbidity detection

**Figure 2:**
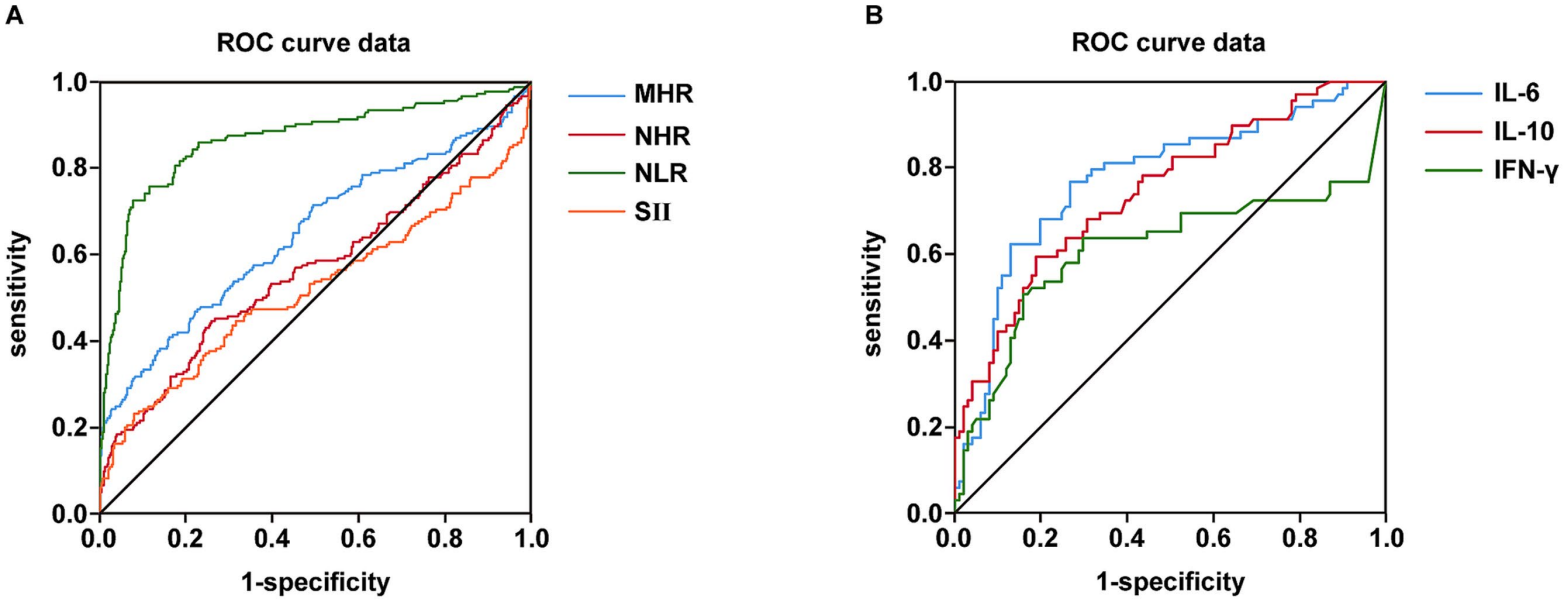
Comparison of inflammatory indexes and cytokines in terms of predicting NAFLD and T2DM using AUC of ROC curve. **(A)** Comparison of MHR, NHR, NLR and SII in terms of predicting NAFLD and T2DM using AUC of ROC curve. **(B)** Comparison of IL-6, IL-10 and IFN-γ in terms of predicting NAFLD and T2DM using AUC of ROC curve.

Figure 2 shows the comparative diagnostic performance of inflammatory indices (MHR, NHR, NLR and SII) and cytokines (IL-6, IL-10 and IFN-γ) through ROC analysis. Moreover, the data distribution of inflammatory indices and cytokines were shown in Supplementary Figure S1. NLR demonstrated superior diagnostic capacity [AUC = 0.868 (0.832–0.904); best cut-off point: 3.28; sensitivity: 72.6%; specificity: 92.4%] relative to MHR (AUC = 0.649), NHR (AUC = 0.568), and SII (AUC = 0.520), with detailed comparisons tabulated in Table 13. Moreover, IL-6 exhibited maximal predictive accuracy among cytokines (AUC = 0.777; best cut-off point: 4.02; sensitivity: 76.8%; specificity: 73.3%) (Table 14). Taken together, NLR and IL-6 were chosen as optimal candidates for subsequent multi parametric diagnostic modeling.

**Table 13:** ROC analysis of MHR, NHR, NLR, and SII.

Outcomes	AUC	95%Cl	Cut-off value	*p-*value	Sensitivity (%)	Specificity (%)
MHR	0.649	0.597–0.701	0.49	**<0.0001**	40.9	84
NHR	0.568	0.514–0.621	4.72	**0.009**	44.6	74.2
NLR	0.868	0.832–0.904	3.28	**<0.0001**	72.6	92.4
SII	0.520	0.463–0.576	822.89	0.446	23.1	92.1

MHR = MONO/HDL-C; NHR = NEU/HDL-C; NLR = NEU/LYM; SII = (NEU×PLT)/LYM. *p*-value <0.05 is considered significant. Bold values represent statistically significant results.

**Table 14:** ROC analysis of IL-6, IL-10, and IFN-γ.

Outcomes	AUC	95%Cl	Cut-off value	*p-*value	Sensitivity (%)	Specificity (%)
IL-6	0.777	0.703–0.850	4.02	**<0.0001**	76.8	73.3
IL-10	0.746	0.671–0.821	3.29	**<0.0001**	59.4	81.2
IFN-γ	0.613	0.515–0.710	0.81	0.013	50.7	84.2

*p*-value <0.05 is considered significant. Bold values represent statistically significant results.

### 3.7 ROC-based combined diagnostic prediction of NLR, TyG, and IL-6 for T2DM-NAFLD comorbidity detection

**Figure 3:**
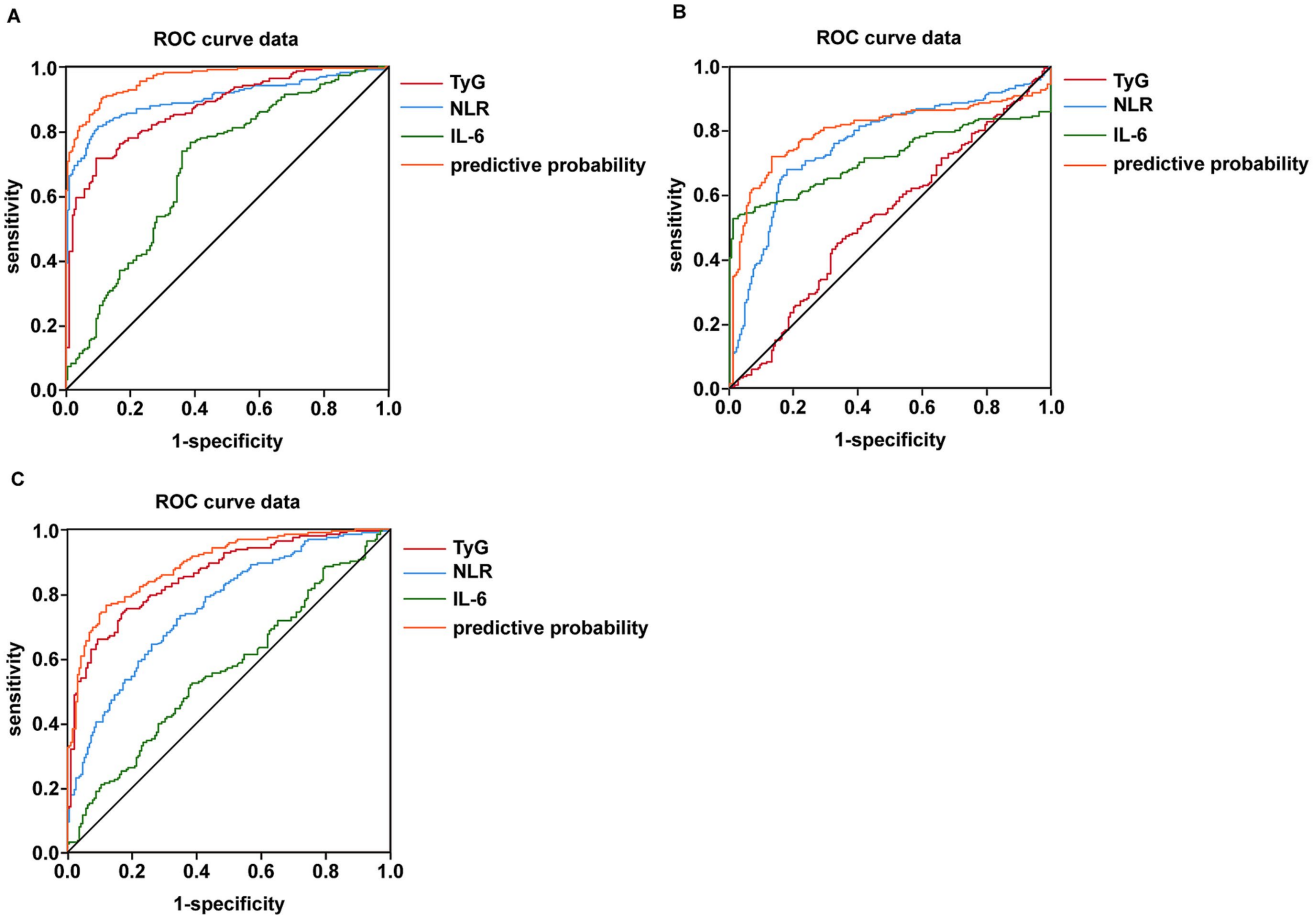
Detection of the predictor value of combined testing of NLR, IL-6, and TyG in terms of predicting NAFLD combine with T2DM using AUC of ROC curve. **(A)** Detection of the predictor value of combined testing of NLR, IL-6 and TyG in terms of predicting NAFLD from healthy individuals using AUC of ROC curve. **(B)** Detection of the predictor value of combined testing of NLR, IL-6 and TyG in terms of predicting NAFLD from T2DM patients using AUC of ROC curve. **(C)** Detection of the predictor value of combined testing of NLR, IL-6 and TyG in terms of predicting T2DM from healthy individuals using AUC of ROC curve.

Based on the results presented in Tables 13, 14 and Figure 2, we selected the most effective inflammatory indicator (NLR) and the cytokines indicator (IL-6) and combined them with the glycohepatic index (TyG) to construct a combined diagnostic model. Subsequently, OR and their corresponding 95% confidence intervals of combined diagnostic prediction are calculated through binary logistic regression analysis. Figure 3 illustrates the performance comparison between combined diagnostic prediction and single diagnosis in identifying NAFLD, as determined through ROC curve analysis. Supplementary Figure S2 shows the data distribution for Figure 3 and Tables 15–17 for details. Table 15 demonstrated that combined diagnostic prediction [AUC = 0.956 (0.945–0.978); best cut-off point: 0.44; sensitivity: 90.1%; specificity: 89.1%] performs best in identifying participants with T2DM-NAFLD comorbidity from healthy individuals compared to a single diagnosis. The combined diagnostic prediction [AUC = 0.802 (0.757–0.848); best cut-off point: 0.5; sensitivity: 72.1%; specificity: 86.9%] also exhibited superior diagnostic capacity relative to a single diagnosis in identifying participants with T2DM-NAFLD comorbidity from T2DM participants (Table 16). Additionally, the combined diagnostic prediction demonstrated better predictive accuracy [AUC = 0.891 (0.859–0.922); best cut-off point: 0.57; sensitivity: 76.4%; specificity: 88.0%] in identifying participants with T2DM from healthy individuals than a single diagnosis (Table 17). In summary, the integrative diagnostic model combined metabolic-inflammatory axes (TyG + NHR + IL-6) and demonstrated superior accuracy in NAFLD detection.

**Table 15:** ROC-based combined diagnostic prediction of TyG, NLR, and IL-6 in control and T2DM-NAFLD comorbidity group.

Outcomes	AUC	95%Cl	Cut-off value	*p-*value	Sensitivity (%)	Specificity (%)
TyG	0.858	0.838–0.905	1.86	**<0.0001**	71.6	90.6
NLR	0.900	0.869–0.931	2.42	**<0.0001**	87.8	95.0
IL-6	0.641	0.641–0.743	4.02	**<0.0001**	76.6	61.5
prediction	0.956	0.945–0.978	0.44	**<0.0001**	90.1	89.1

NLR = NEU/LYM; TyG = LN[TG(mg/dL) × FPG(mg/dL)/2]. *p*-value <0.05 is considered significant. Bold values represent statistically significant results.

**Table 16:** ROC-based combined diagnostic prediction of TyG, NLR, and IL-6 in T2DM and T2DM-NAFLD comorbidity group.

Outcomes	AUC	95%Cl	Cut-off value	*p-*value	Sensitivity (%)	Specificity (%)
TyG	0.532	0.476–0.588	2.26	0.268	45.0	66.5
NLR	0.763	0.715–0.810	3.28	**<0.0001**	68.0	82.2
IL-6	0.717	0.665–0.769	34.27	**<0.0001**	53.6	97.4
prediction	0.802	0.757–0.848	0.50	**<0.0001**	72.1	86.9

NLR = NEU/LYM; TyG = LN[TG(mg/dL) × FPG(mg/dL)/2]. *p*-value <0.05 is considered significant. Bold values represent statistically significant results.

**Table 17:** ROC-based combined diagnostic prediction of TyG, NLR, and IL-6 in control and T2DM group.

Outcomes	AUC	95%Cl	Cut-off value	*p-*value	Sensitivity (%)	Specificity (%)
TyG	0.858	0.821–0.895	1.70	**<0.0001**	75.4	81.8
NLR	0.758	0.711–0.805	1.84	**<0.0001**	76.9	72.5
IL-6	0.563	0.506–0.620	5.52	**0.033**	52.4	61.5
prediction	0.891	0.859–0.922	0.57	**<0.0001**	76.4	88.0

NLR = NEU/LYM; TyG = LN[TG(mg/dL) × FPG(mg/dL)/2]. *p*-value <0.05 is considered significant. Bold values represent statistically significant results.

## 4 Discussion

This research systematically investigated the varying characteristics and clinical implications of inflammatory markers and cytokines in patients with T2DM complicated by NAFLD via a cross-sectional design. The key findings are as follows: patients with T2DM-NAFLD comorbidity present distinct metabolic-inflammatory features; the NLR and IL-6 show remarkable value in differential diagnosis; the combined diagnostic model integrating the TyG, NLR, and IL-6 significantly enhances the recognition accuracy of T2DM-NAFLD comorbidity. These results offer novel clinical evidence for comprehending the role of metabolic inflammation in the comorbid mechanism of T2DM and NAFLD.

### 4.1 Metabolic-inflammatory characteristics of the comorbid population of T2DM-NAFLD

The notable metabolic-inflammatory features exhibited by patients with the comorbidity of T2DM and NAFLD are underpinned by a profound mechanism. This mechanism is embedded in a vicious cycle instigated by IR and intricately intertwined with multiple signaling pathways. The elevation of liver enzymes, dyslipidemia, and the upregulation of systemic inflammation markers, including CRP, FER, NLR, and MHR, as observed in this study, can all be accounted for within this framework.

The core initiating factor is IR. In the setting of T2DM, the insulin signaling pathway, primarily the phosphatidylinositol 3-kinase (PI3K)-Akt axis, is compromised. This leads to unregulated lipolysis in white adipose tissue. A substantial quantity of free fatty acids (FFAs) then inundates the liver, forming the material basis for hepatic steatosis (18–21). This finding aligns with our observation that the TyG index is significantly associated with lipid parameters (Table 2).

The FFAs that enter hepatocytes not only function as lipotoxicity agents but also serve as danger signals to activate the innate immune system. They initiate the downstream inhibitor of nuclear factor kappa B kinase subunit beta (IKKβ)/nuclear factor kappa B (NF-κB) and c-Jun N-terminal kinase (JNK) signaling pathways via pattern recognition receptors such as Toll-like receptor 4 (TLR4) (22, 23).

The nuclear translocation of NF-κB directly promotes the transcription and release of crucial pro-inflammatory factors, including TNF-α, IL-1β, and IL-6 (24, 25). This offers a precise mechanistic account for the significantly elevated IL-6 level and its positive correlation with liver injury markers observed in our study (Table 10).

Simultaneously, FFA-induced ERS and mitochondrial dysfunction further intensify the inflammatory state. ERS indirectly enhances NF-κB activity by activating the inositol-requiring enzyme 1 alpha (IRE1α) and protein kinase RNA-like endoplasmic reticulum kinase (PERK) branches of the unfolded protein response (UPR). Dysfunctional mitochondria generate reactive oxygen species (ROS), which promotes oxidative stress and activates the NLR family pyrin domain-containing 3 (NLRP3) inflammasome. This, in turn, leads to the maturation and secretion of IL-1β and interleukin-18 (IL-18), thereby continuously amplifying the inflammatory cascade (26–29).

Notably, the compensatory increase in IL-10 that we detected might originate from a feedback regulatory mechanism designed to limit excessive inflammatory damage. Nevertheless, under persistent metabolic stress, this protective mechanism is evidently inadequate.

Furthermore, lipid accumulation within hepatocytes and the consequent inflammatory microenvironment exacerbate hepatic insulin resistance via the Janus kinase-signal transducer and activator of transcription (JAK-STAT) pathway, particularly the IL-6-activated JAK2-STAT3 axis, and the feedback of suppressor of cytokine signaling (SOCS) proteins. This process forms a self-reinforcing “metabolic-inflammatory” positive feedback loop (30, 31). Significantly, this loop is not confined to the liver alone; it also impacts whole-body metabolic homeostasis by releasing inflammatory mediators (such as IL-6 and IFN-γ, as detected by our research) and pathogenic lipid species. This phenomenon elucidates the more profound metabolic derangements observed in patients with comorbidities.

In summary, the comorbidity of T2DM and NAFLD is not merely a simple juxtaposition of two distinct diseases. Instead, it represents a persistent, low-grade inflammatory state centered in the liver. This state is initiated by IR and is jointly propelled by multiple signaling pathways, including TLR4/NF-κB, JNK, ERS, and the NLRP3 inflammasome, through intricate cross-talk mechanisms. These findings provide a robust theoretical foundation for considering inflammatory markers as viable diagnostic and therapeutic targets.

### 4.2 The significance of cytokine profiles in disease progression

In Table 4, the observed cytokine profile-specifically, the notable elevation of IL-6, IL-10, and IFN-γ, accompanied by the reduction in IL-2, IL-4, and IL-17- does not represent an isolated biological occurrence. Rather, it serves as the central manifestation of the imbalance within the immune metabolic regulatory network during the progression of T2DM-NAFLD. This particular cytokine pattern profoundly uncovers the molecular essence of the disease’s transition from simple metabolic derangements to chronic inflammation-induced liver injury.

Among these cytokines, the core driving role of IL-6 is of particular significance. IL-6 directly facilitates the synthesis of acute-phase response proteins [such as CRP and FER, which also exhibited corresponding increases in this study (Table 3)] by activating the JAK2/STAT3 signaling pathway within hepatocytes. Simultaneously, it induces the expression of suppressor of cytokine signaling 3 (SOCS3) (32). The latter competitively inhibits the tyrosine phosphorylation of the insulin receptor substrate (IRS), thereby exacerbating hepatic insulin resistance and establishing a vicious cycle.

Furthermore, the continuous activation of STAT3 can also upregulate the activity of hepatic stellate cells (HSCs), which paves the way for the development of liver fibrosis. This offers a potential mechanistic interpretation for the significant elevation of AST and GGT in patients with comorbidities (Table 1) (33).

IFN-γ acts in concert with IL-6. It is predominantly secreted by activated T cells and NK cells. Through its distinct JAK1/STAT1 signaling axis, IFN-γ drives the polarization of liver macrophages (Kupffer cells) toward the pro-inflammatory M1 phenotype and enhances the antigen presentation ability, thereby further amplifying the inflammatory cascade reaction. The positive correlation between IFN-γ and FPG indicates that it may directly participate in the immune inflammatory pathological process of diabetes (Table 12) (34).

The seemingly contradictory elevation of IL-10 in Table 4 interpreted as a compensatory feedback mechanism aiming to limit tissue damage. IL-10 inhibits macrophages from generating TNF-α and IL-12 and promotes their transformation into the anti-inflammatory M2 phenotype by activating the JAK1/STAT3 pathway (sharing some downstream signals with IL-6 but yielding different outcomes in diverse contexts). Nevertheless, in the setting of continuous metabolic stress (such as lipotoxicity and ERS) in patients with T2DM-NAFLD comorbidity, this anti-inflammatory feedback is overpowered by potent pro-inflammatory signals, resulting in the failure to restore immune homeostasis. On the other hand, the reduction in IL-2 and IL-4 indicates the attenuation of helper T cell (Th) function, particularly that of Th2 cells. The substantial decline in IL-17 might reflect a shift in the Th17/Treg balance toward immunosuppression.

This could potentially represent an adaptive reconstruction of the body under persistent inflammatory stress. However, it might also result in an overall deterioration of anti-inflammatory and repair capacities. This finding aligns with the “immune exhaustion” state inferred from the reduction in WBC count, as observed in the study (Table 3). In addition, we supposed that the clinical manifestation of thrombocytopenia was caused by splenomegaly (Supplementary Table S1) in patients with liver disorder (35, 36).

In summary, the dysregulation of the cytokine network in the comorbidity of T2DM and NAFLD is a complex phenomenon. It is predominantly governed by core pro-inflammatory pathways, such as the IL-6/STAT3 and IFN-γ/STAT1 axes. Although there is compensatory activation of anti-inflammatory mechanisms, the overall process ultimately spirals out of control. This intricate network not only directly facilitates the progression of hepatocyte steatosis, inflammatory injury, and fibrosis but also acts as a crucial link connecting systemic insulin resistance to liver-specific pathological alterations.

### 4.3 The correlation network of inflammatory markers and metabolic parameters

Our correlation analysis unveiled an intricate interaction network among inflammatory markers and metabolic parameters. The TyG index, a dependable marker of insulin resistance, exhibited a significant and positive correlation with multiple inflammatory markers, including CRP, FER, MHR, NHR, and NLR (Table 5). This strongly suggests a bidirectional promoting association between insulin resistance and systemic inflammation in the comorbidity of T2DM and NAFLD.

Among diverse inflammatory indexes, NHR demonstrated the most extensive and robust correlations with conventional metabolic parameters. Specifically, it had a high correlation with WBC (*r* = 0.699) and TG levels (*r* = 0.408), indicating that NHR might serve as a sensitive indicator integrating inflammation and dyslipidemia (Table 7). This finding builds upon recent research regarding the value of combined indicators, such as the TyGFI index developed by the Yan Miao team. By integrating metabolic stress and physiological vulnerability, it significantly enhances the capacity to predict cardiovascular risk (37–44).

Notably, we discovered that IL-6 was negatively correlated with WBC (*r* = −0.323), which contradicts the traditional concept of inflammation. We postulate that under the condition of chronic metabolic inflammation, continuous inflammatory stimulation may result in the exhaustion or redistribution of immune cell functions, and this phenomenon merits further investigation.

### 4.4 The liver-spleen axis of non-alcoholic fatty liver disease

Given that the majority of patients with NAFLD exhibit splenomegaly (45), chronic inflammation associated with insulin resistance (46), the phagocytic function and anti-inflammatory effects of the spleen are augmented (47–50). From the perspective of anatomical structure, as the spleen and the liver are connected to visceral fat via the portal vein circulation, the substances secreted by visceral fat directly impact these two organs. Consequently, some scholars have put forward the concept of the hepatosplenic axis. As a crucial organ in the immune cycle, the spleen assumes a dual role in the development of chronic inflammation (51). The spleen is directly implicated in the progression of chronic low-grade inflammation, which in turn precipitates insulin resistance. Similar with our results, the increased expression of IL-6 (52) and IFN-γ (47) were observed in high fat diet mice with enlarged spleen. Moreover, The spleen Ki/V(0) (representing the tissue phosphorylation 18F-fluorodeoxyglucose distribution volume) is correlated with plasma glucose, suggesting the level of insulin sensitivity (45). In comparison to MSG-NO rats, splenectomy in MSG-obese animals can effectively mitigate hyperinsulinemia, enhance insulin sensitivity, and reduce the hypertrophy of adipocytes and islets (53).

However, certain studies have also revealed that the spleen might play a protective role in obesity. Experimental findings have demonstrated that in male Sprague–Dawley rats, regardless of whether they are fed a high-fat diet or a normal diet, splenectomy results in an elevation of serum lipid levels, with the exception of triglycerides and high-density lipoproteins. More significantly, splenectomy markedly accelerates the progression of liver steatosis. Through Western blot analysis and real-time polymerase chain reaction assays, it was discovered that splenectomy not only significantly downregulates the expression level of phosphatase and tensing homolog in the liver but also induces an abnormally high ratio of phosphorylated Akt/Akt in the liver (54). Similarly, researchers observed that after splenectomy in obese mice, the serum interleukin-10 level was further diminished, while the levels of pro-inflammatory cytokines did not decline. These data suggest that spleen-derived interleukin-10 plays a crucial role in the diet-induced inflammatory responses of white adipose tissue and the liver (55).

According with these researches, we plan to establish a database for patients with non-alcoholic fatty liver disease in the future, conduct cohort follow-ups, and track their subsequent progress to continue relevant research, such as “the analysis of the current immune-mediated mechanisms underlying the chronic low-grade inflammation and its subsequent triggering of insulin resistance.”

### 4.5 The value of inflammatory markers in the diagnosis of T2DM-NAFLD

One of the most significant findings of this study was to assess the diagnostic value of multiple inflammatory markers for the comorbidity of T2DM and NAFLD. Receiver operating characteristic (ROC) analysis indicated that the NLR exhibited the highest diagnostic accuracy among blood inflammatory markers [area under the curve (AUC) = 0.868], surpassing the MHR, NHR, and SII (Table 13, Figure 2A). Among cytokines, IL-6 demonstrated the optimal diagnostic performance (AUC = 0.777) (Table 14; Figure 2B). These results suggest that the relatively straightforward and readily obtainable NLR could serve as a valuable tool for primary healthcare institutions to screen the risk of NAFLD in T2DM patients (56).

In line with our findings, numerous studies have corroborated the value of inflammatory markers in the risk assessment of metabolic diseases. The research team led by Dai Dongling discovered that the modified TyG index (incorporating parameters such as waist circumference) had outstanding predictive ability for metabolic-associated fatty liver disease in adolescents (AUC 0.915–0.923). The study by Alam et al. (57) revealed that the fibrosis-4 (FIB-4) index had good performance in the assessment of liver fibrosis in patients with NAFLD complicated by T2DM (AUC = 0.73). Our study further supplements the value of inflammatory markers, especially NLR and IL-6, in the early identification of the simple steatosis stage. Moreover, the comparative analysis of the FIB-4 model have been supplemented. As the results shown in Supplementary Table S2, the AUC for the predictors of combined testing of NLR, IL-6, and TyG is higher than FIB-4 in identifying participants with T2DM-NAFLD comorbidity from T2DM participants (Supplementary Table S2-2) and participants with T2DM from healthy people (Supplementary Table S2-3), further suggesting that the predictors may provide a better predictive capacity in screening patients with T2DM in the early stage or NAFLD in the T2DM patients.

### 4.6 Innovation and clinical significance of the combined diagnostic model

The most groundbreaking discovery of this study lies in the establishment of a composite diagnostic model (TyG + NHR + IL-6) that integrates metabolism, inflammation, and cytokines. This model exhibited outstanding diagnostic performance in discriminating patients with T2DM complicated by NAFLD from those with simple T2DM, with an area under the receiver operating characteristic curve (AUC) of 0.802 (Table 16; Figure 3B). Moreover, it demonstrated even superior performance when differentiating comorbid patients from healthy controls, achieving an AUC of 0.956 (Table 15; Figure 3A).

This multi-dimensional diagnostic approach is highly congruent with the concept of contemporary precision medicine, which aims to enhance disease identification capabilities by integrating biomarkers from diverse pathophysiological pathways. Our method shares similarities with the research concept of the metabolic-inflammatory subtypes described above; however, it is more clinically applicable and feasible. In contrast to subtype classification that necessitates intricate metabolomics analysis, our composite model is founded on routine clinical indicators, rendering it more amenable to promotion in clinical practice.

Furthermore, compared with the TyGFI index developed by the Yan Miao team, our model specifically targets the identification of T2DM-NAFLD comorbidity, potentially offering a more precise screening tool for this particular patient population.

According to our results and in line with the 2024 ADA guidelines, we suggest for the annual implementation of a systematic screening protocol in high-risk populations, such as T2DM patients. This protocol usually begins with the FIB-4 index, followed by confirmatory testing with transient electrography (e.g., Fibro Scan) for those with indeterminate or elevated scores. To address the limitations of the secondary consumption and complex inspection process, our study proposes an alternative method utilizing a novel combination predictive marker of NLR, IL-6 and TyG. Combination predictive marker with a value greater than 0.44 may be considered high risk (Tables 15–17). The management for high-risk patients must be comprehensive, which including grounded in lifestyle modifications (targeted weight loss, dietary control, exercise) and augmented by individualized pharmacological regimens. This integrated strategy coupled with regular 3- to 6-month follow-ups to monitor key parameters is essential for effective clinical management.

### 4.7 Limitations and future perspectives

This investigation encompasses several limitations. Firstly, the cross-sectional design employed herein precludes the determination of causal relationships among the observed associations. In the future, prospective cohort studies are imperative to validate the predictive utility of these indicators. Secondly, the diagnosis of non-alcoholic fatty liver disease (NAFLD) is predicated on clinical characteristics rather than the gold standard of liver biopsy. This approach may potentially overlook subclinical cases. Thirdly, not all potential confounding factors, such as dietary patterns, physical activity levels, and gut microbiota composition, which could influence the inflammatory state, were comprehensively evaluated. Finally, the study population was sourced from a single center. Consequently, the external validity of the findings necessitates verification through multi-center investigations.

Future research endeavors should center on the following aspects:

1. Validating the predictive value of the combined diagnostic model within a prospective cohort;
2. Probing into the role of these indicators in monitoring disease progression and treatment response;
3. Conducting in-depth mechanistic explorations of the molecular pathways underlying the identified inflammatory characteristics, such as the IKKε-NF-κB pathway and the Nrf2-HDAC axis;
4. Formulating personalized intervention strategies grounded in metabolic-inflammatory characteristics.

## 5 Conclusion

In conclusion, the research demonstrates that NLR, IL-6, and TyG are important reference indexes for identifying the incidence in patients with T2DM-NAFLD comorbidity from healthy individuals and patients with T2DM. The integrative diagnostic model (TyG + NLR + IL-6) also demonstrates superior accuracy in NAFLD detection, substantiating its clinical translation potential for early metabolic dysfunction identification and treatment.

## Data Availability

The original contributions presented in the study are included in the article/Supplementary material, further inquiries can be directed to the corresponding authors.
