# Supplementary material for: The inflammatory index and cytokines are associated with non-alcoholic fatty liver disease in type 2 diabetes mellitus
Source: Front Med (Lausanne). 2025 Nov 26;12:1659998. doi: 10.3389/fmed.2025.1659998 (PMC12685878; doi:10.3389/fmed.2025.1659998)
Supplement: Supplementary file 1 [file Supplementary_file_1.docx]

Table S1. Spleen characteristics of the participants.

| Outcomes | Control  (n=40) | T2DM  (n=40) | T2DM+NAFLD  (n=40) | *p^1^-*value | *p^2^-*value | *p^3^-*value |
| --- | --- | --- | --- | --- | --- | --- |
| Mean cumulative spleen ROI (cm^2^)  mean (s.d.) | 17.61  (2.66) | 21.69  (3.77) | 70.46  (9.85) | **<0.0001** | **<0.0001** | **<0.0001** |

Data were shown as means ± SD. *p^1^*: Control group vs. T2DM group; *p^2^*: Control group vs. T2DM+NAFLD group; *p^3^*: T2DM group vs. T2DM+NAFLD group. *p*-value < 0.05 is considered significant. Bold values represent statistically significant results.

TABLE S2-1. ROC-based combined diagnostic prediction of TyG, NLR, and IL-6 and FIB-4 in control and T2DM-NAFLD comorbidity group

| Outcomes | AUC | 95%Cl | Cut-off value | *p-*value | Sensitivity（%） | specificity（%） |
| --- | --- | --- | --- | --- | --- | --- |
| prediction | 0.956 | 0.945-0.978 | 0.44 | **<0.0001** | 90.1 | 89.1 |
| FIB-4 | 0.984 | 0.963-1.000 | 1.91 | **<0.0001** | 97.2 | 97.9 |

NLR=NEU/LYM; TyG=LN(TG[mg/dL]×FPG[mg/dL]/2); FIB-4=(Age/year×AST)÷(PLT×√ALT). *p*-value < 0.05 is considered significant. Bold values represent statistically significant results.

TABLE S2-2. ROC-based combined diagnostic prediction of TyG, NLR, and IL-6 and FIB-4 in T2DM and T2DM-NAFLD comorbidity group

| Outcomes | AUC | 95%Cl | Cut-off value | *p-*value | Sensitivity（%） | specificity（%） |
| --- | --- | --- | --- | --- | --- | --- |
| prediction | 0.802 | 0.757-0.848 | 0.5 | **<0.0001** | 72.1 | 86.9 |
| FIB-4 | 0.763 | 0.715-0.810 | 2.55 | **<0.0001** | 92.5 | 94.1 |

NLR=NEU/LYM; TyG=LN(TG[mg/dL]×FPG[mg/dL]/2);FIB-4=(Age/year×AST)÷(PLT×√ALT). *p*-value < 0.05 is considered significant. Bold values represent statistically significant results.

TABLE S2-3. ROC-based combined diagnostic prediction of TyG, NLR, and IL-6 and FIB-4 in control and T2DM group

| Outcomes | AUC | 95%Cl | Cut-off value | *p-*value | Sensitivity（%） | specificity（%） |
| --- | --- | --- | --- | --- | --- | --- |
| prediction | 0.891 | 0.859-0.922 | 0.57 | **<0.0001** | 76.4 | 88.0 |
| FIB-4 | 0.747 | 0.689-0.805 | 0.85 | **<0.0001** | 75.7 | 68.3 |

NLR=NEU/LYM; TyG=LN(TG[mg/dL]×FPG[mg/dL]/2);FIB-4=(Age/year×AST)÷(PLT×√ALT). *p*-value < 0.05 is considered significant. Bold values represent statistically significant results.


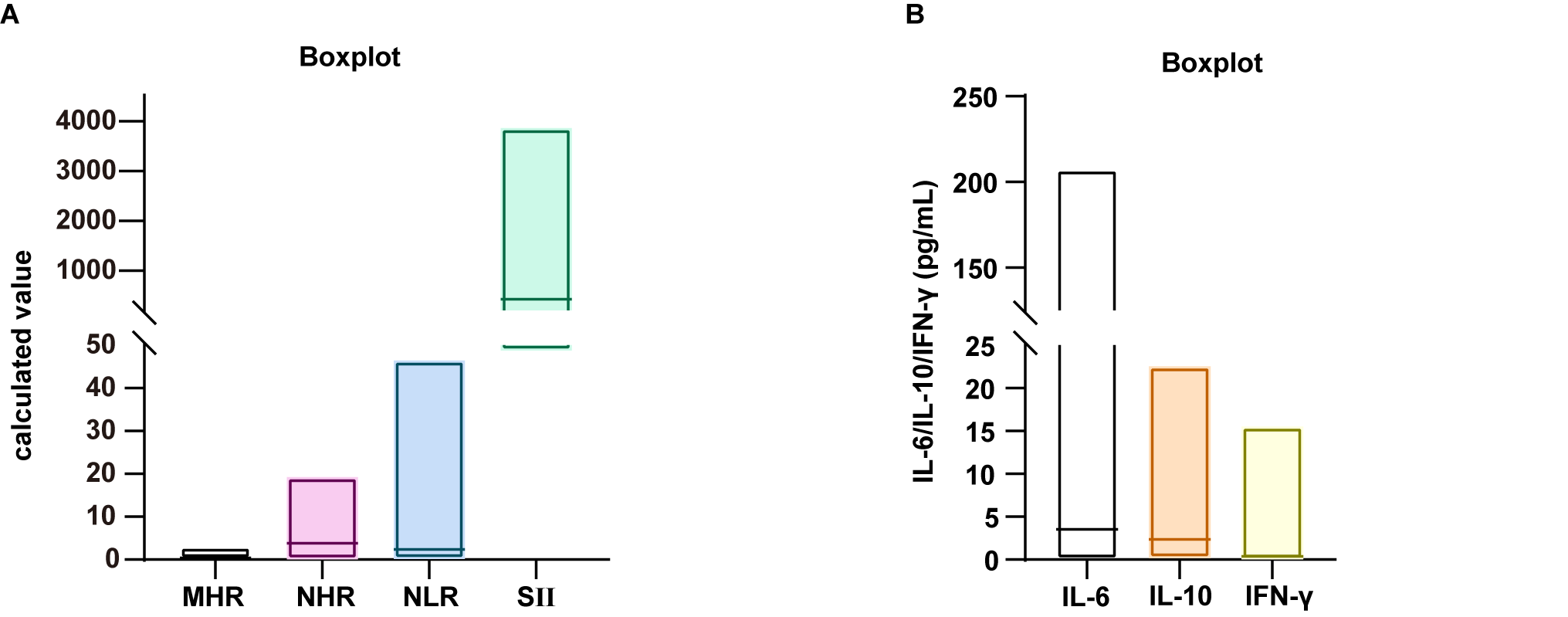


**Supplemental Figure 1. Data boxplot of Figure 2.**

A: Data boxplot of inflammatory indexes (MHR, NHR, NLR and SⅡ). B: Data boxplot of cytokines (IL-6, IL-10 and IFN-γ).


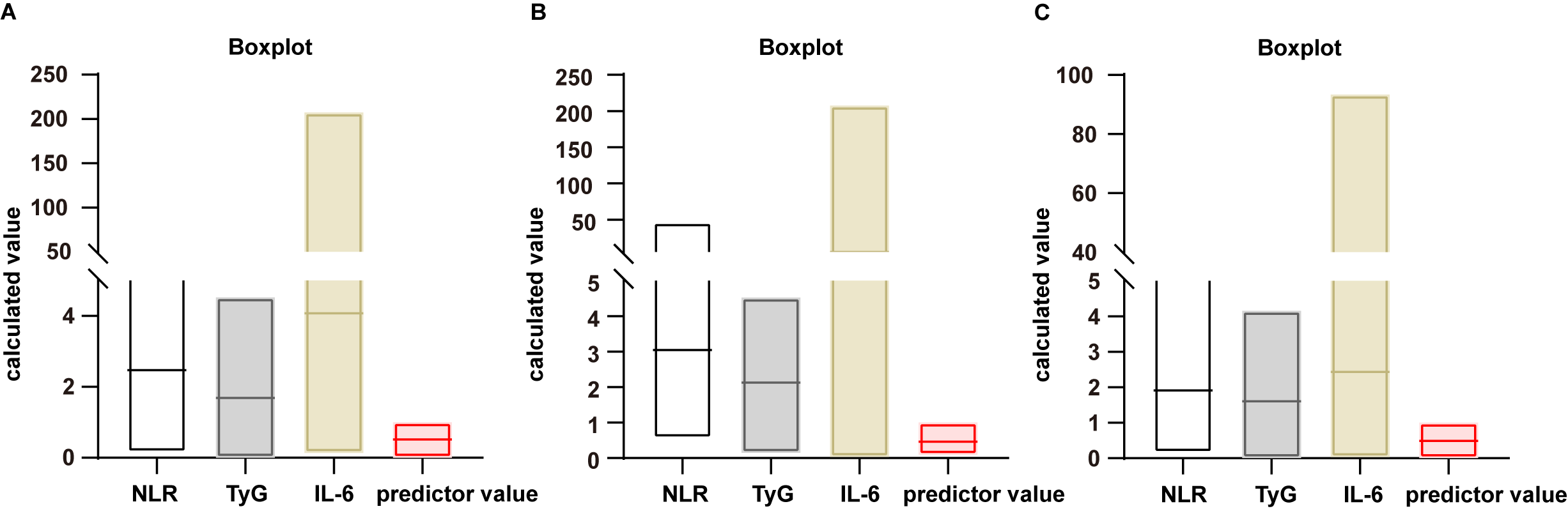


**Supplemental Figure 2. Data boxplot of Figure 3.**

A: Data boxplot of NLR, IL-6, TyG and the predictor value of combined testing of NLR, IL-6 and TyG in control and T2DM-NAFLD comorbidity group. B: Data boxplot of NLR, IL-6, TyG and the predictor value of combined testing of NLR, IL-6 and TyG in T2DM and T2DM-NAFLD comorbidity group. C: Data boxplot of NLR, IL-6, TyG and the predictor value of combined testing of NLR, IL-6 and TyG in control and T2DM group.
